# Hepatoid adenocarcinoma of the stomach with ideal response to neoadjuvant chemo-immunotherapy: a case report

**DOI:** 10.3389/fimmu.2024.1496342

**Published:** 2025-01-08

**Authors:** Linchuan Li, Dexu Zhang, Jiankang Zhu, Guangyong Zhang

**Affiliations:** ^1^ Department of General Surgery, The First Affiliated Hospital of Shandong First Medical University & Shandong Provincial Qianfoshan Hospital, Shandong, China; ^2^ Department of General Surgery, Shandong Provincial Qianfoshan Hospital, The First Affiliated Hospital of Shandong First Medical University, Laboratory of Metabolism and Gastrointestinal Tumor, Shandong, China; ^3^ Shandong Provincial Qianfoshan Hospital, Shandong University, Shandong, China

**Keywords:** hepatoid adenocarcinoma of stomach, immunotherapy, chemotherapy, programmed cell death-ligand 1, laparoscopic gastrectomy, gastric cancer

## Abstract

Hepatoid adenocarcinoma of the stomach (HAS) is a rare subtype of gastric cancer characterized by histological features resembling hepatocellular carcinoma. Surgical intervention remains the preferred treatment modality for eligible patients. However, the efficacy of neoadjuvant therapy and alternative treatment regimens has been found to be suboptimal. Consequently, due to the high metastatic potential and unfavorable biological behavior of HAS, the prognosis for affected patients is exceedingly poor. We present a case involving a 64-year-old male diagnosed with advanced HAS, who demonstrated significant antitumor responses following a preoperative regimen of chemotherapy combined with immunotherapy, specifically utilizing oxaliplatin, S-1, and sintilimab. Over a 2-month period of neoadjuvant therapy, the patient’s serum α-fetoprotein level significantly decreased from 52,951.56 ng/mL to 241.04 ng/mL. Computed tomography scans revealed substantial tumor regression. Subsequent radical surgical intervention confirmed significant tumor shrinkage, with no evidence of lymph node metastasis upon pathological examination. This is the first report of chemotherapy combined with sintilimab in the treatment of gastric hepatoid adenocarcinoma, which may provide novel insights into the therapeutic strategy for HAS.

## Introduction

Hepatoid adenocarcinoma of the stomach (HAS) is a rare subtype of gastric cancer featuring adenoid and hepatocyte differentiation, which is identical to hepatocellular carcinoma. HAS was first reported by Ishikura et al. in 1985, as a specificα-fetoprotein (AFP)-producing gastric cancer (1). HAS has been described in multiple organs, such as the stomach, pancreas, colon, and ovaries, of which the stomach is the most common site (2). HAS usually occurs in older males, with an average age at onset of approximately 60 years old (3). HAS accounts for only 0.3% to 1% of all kinds of gastric cancer (4, 5). The most common onset area of HAS is the gastric antrum, while it is rarely found in the cardia and gastric fundi (6).

Thus far, accurate diagnosis remains challenging due to the typically small proportion of the gastric region affected by HAS, which may complicate endoscopic biopsy procedures. With similar clinical features as common types of gastric cancer, HAS is typically latent and lacks specific clinical symptoms, which may make early diagnosis difficult (6).

Surgical intervention is commonly employed as the primary treatment strategy for HAS. Due to the high metastatic potential and adverse biobehavioral characteristics, the prognosis of HAS remains extremely poor. According to various studies, the 5-year survival rate ranges from 8.3% to 34.0% (7–10). Significant challenges persist in the development of appropriate and effective treatments for HAS. Despite many patients undergoing surgical treatment, the prognosis still appears poor (11). Although adjuvant chemotherapy and neoadjuvant chemotherapy have been employed for patients with HAS, it remains a challenge to determine a standard and effective treatment regimen, for either effective drugs or drug combinations (12, 13). However, herein, we report a case of a 64-year-old man who received preoperative chemotherapy and immunotherapy, followed by radical surgery, with satisfactory outcomes.

## Case description

**Figure 1:**
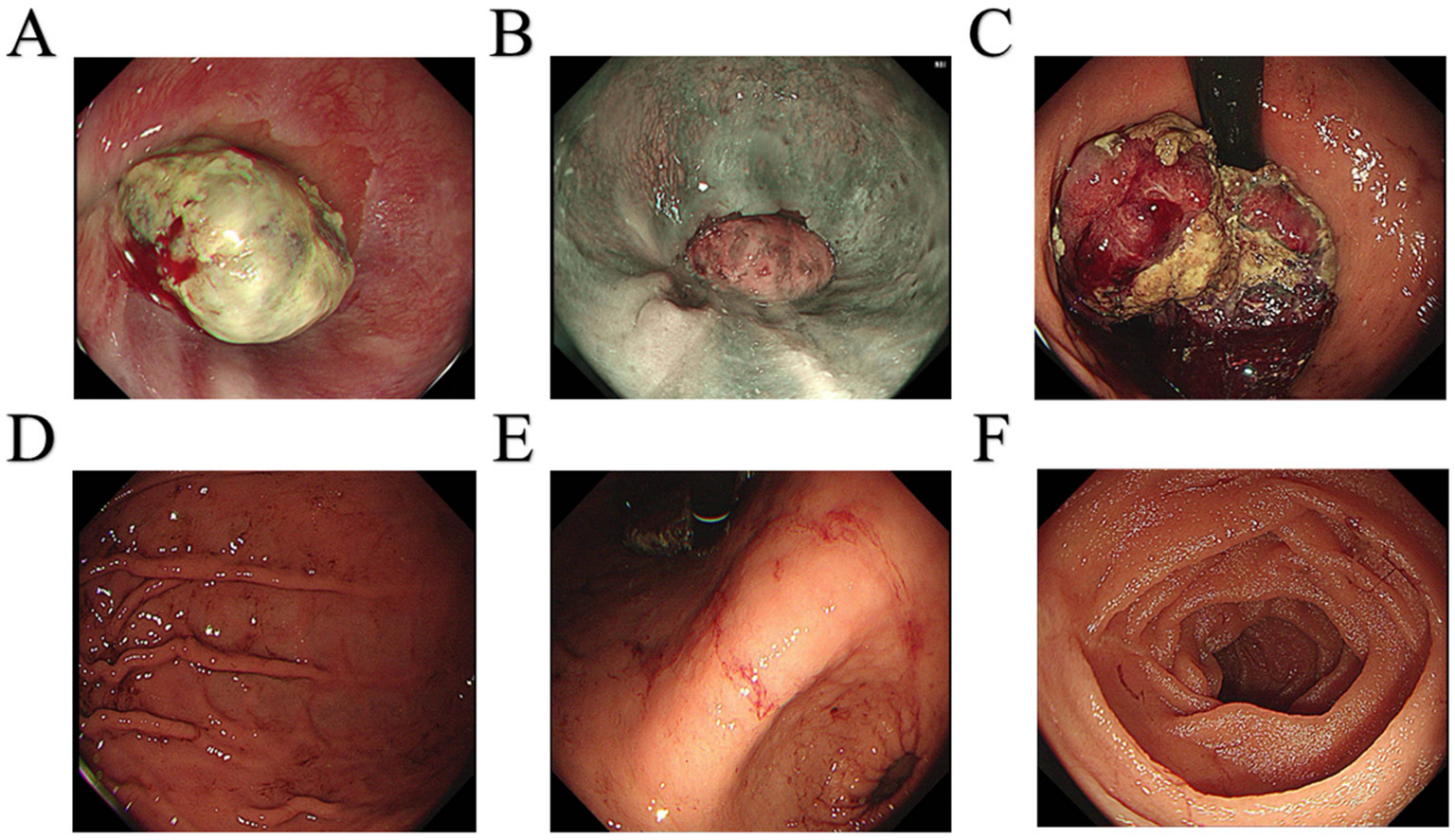
Gastroscopy images. **(A)** Dentate line. **(B)** Esophagus. **(C)** Fundus of the stomach. **(D)** Gastric body. **(E)** Gastric angle. **(F)** Duodenum.

**Figure 2:**
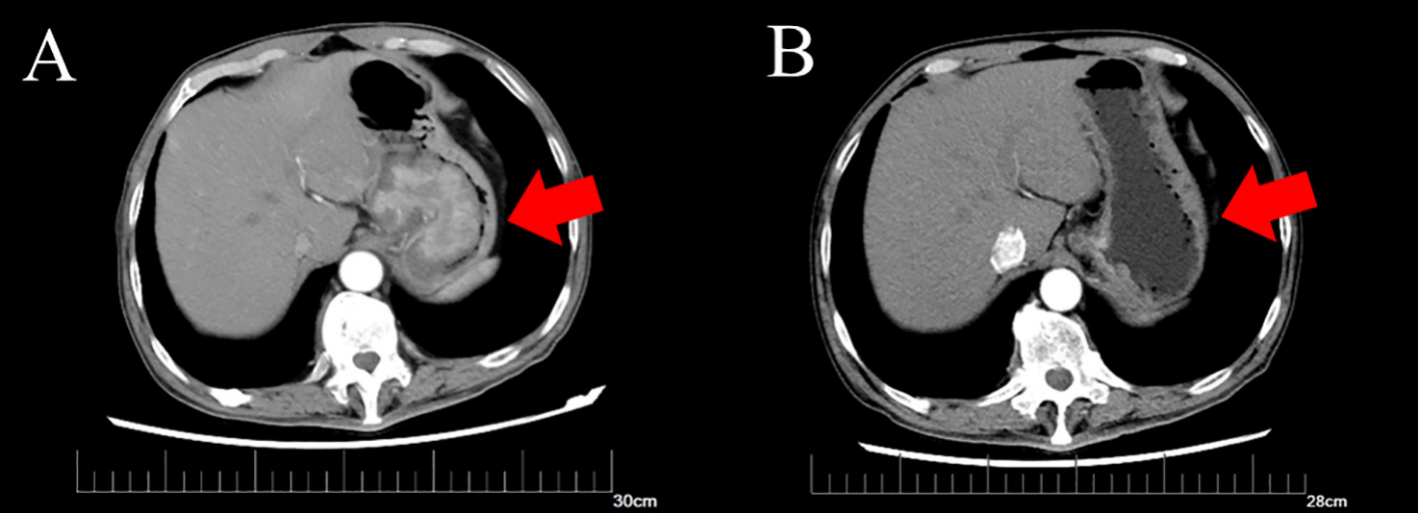
Computed tomography of gastric tumor before and after neoadjuvant therapy. **(A)** CT of the irregular mass with dimensions of 6.3*5.0 cm before neoadjuvant therapy. **(B)** CT of irregular thickening on the gastric fundus and the range is obviously regressed compared to before.

A 64-year-old male patient was admitted to the hospital with a 1-month history of fatigue and unspecified gastrointestinal discomfort. The patient claimed no symptoms of abdominal pain, nausea, vomiting, reflux, or dysphagia. The patient was treated with omeprazole tablets to suppress gastric acid secretion prior to admission, with no significant relief of symptoms. The patient claimed no family history of gastrointestinal tract cancer. Upon admission, laboratory tests revealed a hemoglobin level of 55.0 g/L, indicating anemia. Tumor markers for the digestive tract showed a significant elevation in serum AFP levels, measured at 52,951.56 ng/mL (reference range: 0-8.78 ng/mL), and a slight elevation in carcinoembryonic antigen, measured at 8.94 ng/mL (reference range:0-5.0 ng/mL). Additionally, stool analysis tested positive for occult blood. Gastroscopy indicated the presence of a large, irregular mass measuring approximately 4.5 cm in diameter located in the fundus of the cardia (**Figure 1**). The surface of the mass was uneven, brittle, and prone to bleeding. *Helicobacter pylori* testing was negative. Pathological analysis confirmed a diagnosis of poorly differentiated adenocarcinoma of the gastric cardia. Immunohistochemical results revealed positive expression of the carcinoembryonic protein alpha-fetoprotein (AFP), spalt-like transcription factor 4(SALL4), and hepatocyte-specific antigen. The expression of human epidermal growth factor receptor 2 (Her2) was negative. In conjunction with the aforementioned findings, the patient was ultimately diagnosed with gastric adenocarcinoma that produced AFP, with a subset identified as hepatoid adenocarcinoma. Contrast-enhanced computed tomography (CT) revealed an irregular lobulated mass with a diameter of approximately 6 cm, situated on the inferior curvature of the gastric fundus, exhibiting distinct heterogeneous enhancement. Additionally, multiple enlarged lymph nodes were observed surrounding the stomach and within the lesser omental sac, all demonstrating uniform enhancement (**Figure 2A**). Chest CT showed no signs of metastasis.

Following a multidisciplinary discussion involving the Imaging Department, Medical Oncology Department, and Gastrointestinal Surgery Department, the patient’s TNM stage was determined to be T4N1-2Mx. Neoadjuvant therapy was initially recommended. An additional immunohistochemical test for programmed cell death-ligand 1(PD-L1) indicated that positive tumor cells and tumor-associated immune cells accounted for approximately 1%. A test for microsatellite instability showed no deficient mismatch repair. Therefore, the prescribed neoadjuvant therapy regimen comprised chemotherapy and immunotherapy and included oxaliplatin and S-1, in combination with sintilimab. The patient subsequently underwent three cycles of neoadjuvant therapy, resulting in significant tumor and lymph node regression as shown by enhanced CT (**Figure 2B**). After ruling out surgical contraindications, a laparoscopic total gastrectomy with lymph node dissection was successfully performed. Intraoperatively, it was observed that the tumor was located in the cardia and had not penetrated the serous membrane. Resected gastric and lymph node specimens were submitted for subsequent pathological examination.

**Figure 3:**
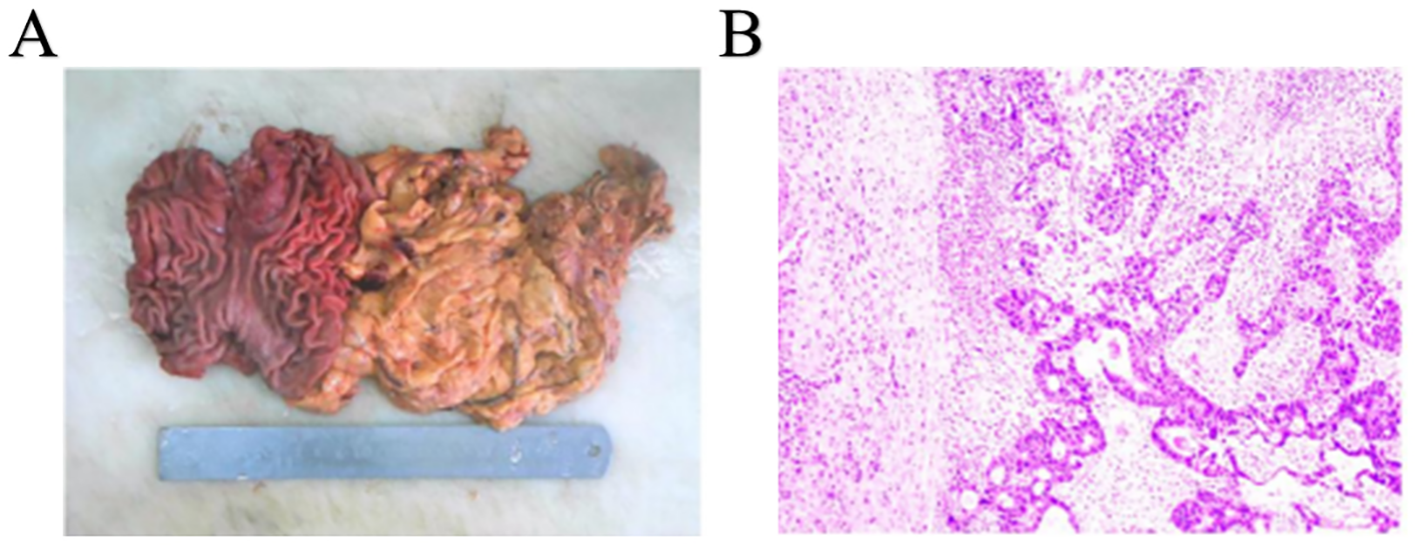
Completely resected tumor and histopathological findings. **(A)** Complete resected tumor. **(B)** histopathological findings of gastric tumor.

Upon gross examination, a 3.8 x 1.5 cm infiltrating ulcerative mass was identified near the cardia, following an incision along the greater curvature. Sectioning of the tumor revealed a tough brown and gray mass that had invaded the muscle layer, lacking clear demarcation from the surrounding structures (**Figure 3A**). Mucosal erosion was observed around the tumor, with acute and chronic inflammation and hyperplasia of the fibro granulomatous tissue, which was consistent with chemotherapy changes. None of the perigastric lymph nodes exhibited metastatic involvement. Subsequent immunohistochemical analysis demonstrated positivity for Her2, while P63 and CK5/6 were negative (**Figure 3B**). The final diagnosis was AFP-producing gastric adenocarcinoma with partial hepatoid adenocarcinoma differentiation.

**Figure 4:**
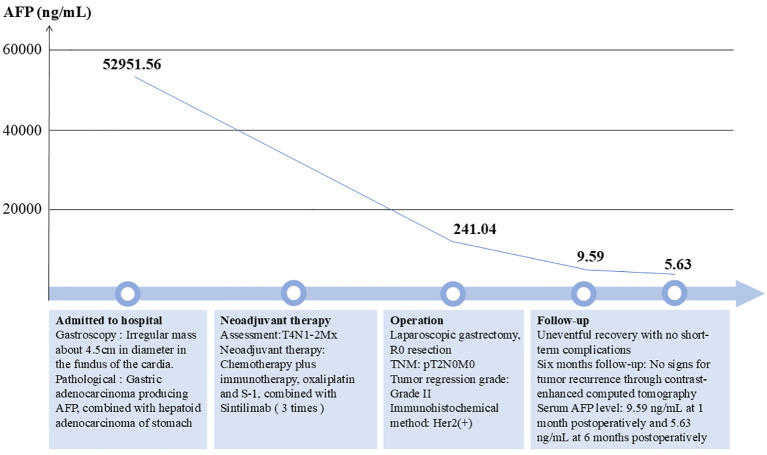
The whole treatment chart with the AFP trend.

The patient experienced an uneventful postoperative recovery, with no short-term complications, and was discharged from the hospital on the ninth postoperative day. An iodine contrast study of the upper digestive tract indicated optimal recovery of the anastomotic site, with no evidence of leakage or stenosis. During the follow-up period, the patient’s AFP levels exhibited a significant decline, decreasing from 52,951.56 ng/mL prior to surgery to 241.04 ng/mL following preoperative neoadjuvant therapy, and further reducing to 9.59 ng/mL 1 month after surgery. At the 6-month follow-up, the patient had excellent recovery and there was no evidence of recurrence in enhanced CT of the chest and abdomen. The serum AFP level was 5.63 ng/mL (**Figure 4**).

## Discussion

Since hepatoid adenocarcinoma was first described in 1985, there have been multiple reports of cases affecting various organs, including gastrointestinal organs such as the esophagus, pancreas, and appendix; urogenital organs such as ovaries, the uterus, and adrenal glands; and other organs such as the lungs (14–20). Among these, the stomach is the most common site, and as such, hepatoid adenocarcinoma is classified as a rare type of gastric cancer. Recent studies from Asia have reported a HAS incidence of 0.17% to 0.36% (21, 22). The origin and pathogenesis of HAS remain uncertain. Previous research has suggested that HAS may originate from the endoderm, which develops from adenocarcinoma with an intestinal phenotype during embryonic development (23). A recent study investigating the origin of HAS demonstrated that both the adenocarcinomatous and hepatocellular-like components of HAS originate from a monoclonal pluripotent precursor cell (24).

The molecular characteristics of HAS remain poorly understood. However, a genetic analysis conducted on 42 patients with HAS identified TP53, CEBPA, RPTOR, WISP3, MARK1, and CD3EAP as genes with high-frequency mutations, exhibiting mutation rates ranging from 10% to 30% (25). These mutated genes may contribute to the enrichment of the HIF-1 signaling pathway and also signaling pathways regulating stem cell pluripotency in HAS.

The manifestation of HAS is typically latent and lacks specific clinical symptoms, resembling common types of gastric cancer, thereby complicating early diagnosis. The initial clinical presentation often includes non-specific upper abdominal discomfort (26). Consequently, the accurate and reliable identification of HAS remains a significant challenge. CT is considered an optimal choice for the diagnosis of HAS, often revealing a thickened gastric wall and invasion of the peritumoral fatty space, accompanied by continuous enhancement (27, 28). However, some studies have suggested that the diagnostic value of CT for HAS may be limited, because it may not show significant anatomic abnormalities at the site of the primary tumor (29). Recently, some research studies have highlighted the significance of positron emission tomography (PET)/CT in diagnosing and differentiating HAS accurately, which needs confirmation for further application (30).

HAS exhibits similar histological features as hepatocellular carcinoma, which is characterized by the concurrent presence of adenocarcinoma and hepatoid components in pathological examinations (31). Furthermore, through immunohistochemical tests, HAS features the positive expression of AFP, GPC-3, and SALL4 (32). As the most prevalent subtype of AFP-producing gastric cancers, HAS is distinguished by its heightened invasive and metastatic potential, which is associated with an extremely poor prognosis (33). Furthermore, numerous studies have investigated the correlation between AFP expression levels and patient prognosis, though the findings remain contentious. A study conducted in China demonstrated that elevated serum AFP levels serve as an independent prognostic factor in gastric cancer, correlating with poorer outcomes, as evidenced by an analysis of 1,286 gastric cancer patients (34). Another study revealed that the 1-year survival rates for patients with AFP levels ≤20 ng/ml, ≤300 ng/ml, and >300 ng/ml were 75.2%, 46.7%, and 15.4%, while the 5-year survival rates were 45.8%, 17.8%, and 0%, respectively (35). Furthermore, Yakun Wang and colleagues reported that a preoperative serum AFP level of ≥500 ng/mL was strongly associated with poor overall survival (25). In contrast, a Japanese study found no correlation between preoperative serum AFP levels and survival outcomes, although postoperative elevations in serum AFP were frequently indicative of tumor recurrence (10).

At present, there are no specialized treatments available for HAS, and the most common therapeutic approach remains radical surgery, which is also the conventional treatment for the more typical forms of gastric cancer. For patients with advanced-staged HAS that is not amenable to surgical resection, systemic chemotherapy, including neoadjuvant treatment, may represent a potential therapeutic option (3). Neoadjuvant therapy has the benefits of reducing the tumor burden and improving overall survival (36). However, there remains no established optimal and effective standard for such treatments. Genomic analysis of HAS has demonstrated elevated drug transport activity and increased expression of drug-resistance-related genes compared to more typical forms of gastric cancer, indicating that conventional chemotherapy may not be an ideal treatment approach (24). Although studies have demonstrated the clinical benefit of neoadjuvant chemotherapy for other forms of gastric cancer, its therapeutic efficacy for HAS still remains a subject of debate. Certain studies have suggested that FOLFOX could serve as a potential postoperative treatment for HAS, whereas other studies have reported less favorable outcomes (12, 37, 38). In our study, based on standard therapy for advanced gastric cancer, we applied the SOX protocol and found a remarkable curative effect on tumor reduction. Furthermore, due to the R0 resect of the tumor, with a TNM stage of T2N0M0, this patient received careful follow-up without postoperative chemotherapy. Through 6 months of follow-up, there was no evidence of tumor recurrence based on enhanced CT scans and serum tests for AFP level. Currently, immunotherapy is infrequently applied for HAS. A previous case report indicated that sintilimab exhibited a satisfactory therapeutic effect in a patient with advanced lung hepatoid adenocarcinoma (39). However, there have been no reports concerning its efficacy in the treatment of HAS. Our study represents the first report to demonstrate the significant efficacy of sintilimab in the treatment of HAS, which demonstrated promising therapeutic effects.

Our study suggests that the combination of chemotherapy and immunotherapy, such as sintilimab, may yield significant outcomes and could serve as a potential adjuvant treatment option for HAS. These findings may contribute valuable insights for the development of treatment strategies for patients with advanced HAS and underscore the importance of molecular diagnosis in informing treatment decisions.

## Conclusion

HAS is an uncommon subtype of gastric cancer characterized by a poor prognosis. Metastasis to the lymph nodes and distant organs, especially the liver, is often present at diagnosis, which poses a huge challenge for treatment and less favorable therapeutic efficacy. The standard treatment protocol for HAS remains undefined. This case report suggests that a combination of SOX chemotherapy and immunotherapy, specifically sintilimab, may represent an effective therapeutic option for advanced HAS. Further in-depth investigations and prolonged follow-ups of related cases are necessary to provide more robust evidence for the treatment of HAS.

## Data Availability

The raw data supporting the conclusions of this article will be made available by the authors, without undue reservation.
